# *Mycoplasma iowae*: relationships among oxygen, virulence, and protection from oxidative stress

**DOI:** 10.1186/s13567-015-0170-7

**Published:** 2015-03-21

**Authors:** Rachel E Pritchard, Mitchell F Balish

**Affiliations:** Department of Microbiology, Miami University, Oxford, OH 45056 USA; Present address: Division of Natural Sciences and Mathematics, Kentucky Wesleyan College, Owensboro, KY 42301 USA

## Abstract

The poultry-associated bacterium *Mycoplasma iowae* colonizes multiple sites in embryos, with disease or death resulting. Although *M. iowae* accumulates in the intestinal tract, it does not cause disease at that site, but rather only in tissues that are exposed to atmospheric O_2_. The activity of *M. iowae* catalase, encoded by *katE*, is capable of rapid removal of damaging H_2_O_2_ from solution, and *katE* confers a substantial reduction in the amount of H_2_O_2_ produced by *Mycoplasma gallisepticum katE* transformants in the presence of glycerol. As catalase-producing bacteria are often beneficial to hosts with inflammatory bowel disease, we explored whether *M. iowae* was exclusively protective against H_2_O_2_-producing bacteria in a *Caenorhabditis elegans* model, whether its protectiveness changed in response to O_2_ levels, and whether expression of genes involved in H_2_O_2_ metabolism and virulence changed in response to O_2_ levels. We observed that *M. iowae* was in fact protective against H_2_O_2_-producing *Streptococcus pneumoniae*, but not HCN-producing *Pseudomonas aeruginosa*, and that *M. iowae* cells grown in 1% O_2_ promoted survival of *C. elegans* to a greater extent than *M. iowae* cells grown in atmospheric O_2_. Transcript levels of an *M. iowae* gene encoding a homolog of *Mycoplasma pneumoniae* CARDS toxin were 5-fold lower in cells grown in low O_2_. These data suggest that reduced O_2_, representing the intestinal environment, triggers *M. iowae* to reduce its virulence capabilities, effecting a change from a pathogenic mode to a potentially beneficial one.

## Introduction

H_2_O_2_ is a dangerous reactive oxygen species (ROS) involved in both pathogenesis and defense against infectious agents. Bacteria may be exposed to multiple sources of H_2_O_2_ during infection, including macrophages, which produce a variety of ROS that damage and degrade bacteria. A variety of bacterial pathogens can also produce H_2_O_2_ as a means of causing damage to host tissues [1,2], and some disease states stem from exposure of tissues to ROS including H_2_O_2_. In particular, in inflammatory bowel disease (IBD), a growing problem in humans in developed countries with a prevalence of 10-20% [3], a direct correlation between increased ROS production and damage to gut epithelial cells has been reported [4-7].

The enzyme catalase catalyzes degradation of H_2_O_2_ [8]. The production of catalase by microbes can benefit both the microorganisms themselves and a host organism with which they associate. Catalase can protect catalase-producing bacteria from environmental H_2_O_2_ and prolong infection. As for the host, several reports have examined the ability of catalase-expressing probiotic bacteria to decrease IBD symptoms [9]. In a murine trinitrobenzenesulfonic acid-induced Crohn’s disease model, administration of *Lactobacillus casei* engineered to express catalase results in faster recovery from initial weight loss, increased gut enzyme activity, and decreased intestinal inflammation as compared with mice infected with wild-type or no bacteria [10]. Similar bacteria decrease cecal and colonic inflammation in mice treated with dextran sodium sulfate to induce moderate colitis [11].

*Mycoplasma iowae* is a catalase-positive bacterium that infects poultry animals, primarily turkeys but also occasionally chickens [12,13]. The most common outcome of naturally-occurring *M. iowae* infection in turkeys is late embryo mortality, with a 2-5% reduction in hatchability, and leg abnormalities in offspring [14-16]. Symptoms commonly associated with experimental infection include airsacculitis, stunting, poor feathering, and leg and joint problems [14,16]. The *M. iowae* catalase gene, *katE*, confers both catalase activity and a significant reduction in H_2_O_2_ production upon *Mycoplasma gallisepticum*, a robust H_2_O_2_ producer that elaborates no other known toxins [13,17,18]. It is unclear how *M. iowae* causes disease, but in addition to its attachment organelle-mediated adherence and motility functions [19], its genome encodes potential virulence factors, including two closely linked genes encoding proteins similar to *Mycoplasma pneumoniae* CARDS toxin [13], an ADP-ribosylating toxin that is associated with many of the symptoms of *M. pneumoniae* infection [20-22].

Interestingly, *M. iowae* colonizes a variety of body sites with a range of O_2_ concentrations. It has been isolated from the cloacae of healthy poults and mature turkeys [23,24], but also infects the chorioallantoic membrane and a variety of organs in embryos [25], which accounts for the morbidity and mortality associated with *M. iowae*. Significantly, *M. iowae* also has a pronounced tendency to colonize the gut with no ill effects. Following yolk sac inoculation of eight-day-old turkey embryos, *M. iowae* can be detected in the small intestines, with bacteria most often attaching to microvilli [25]. Oral inoculation of day-old poults with *M. iowae* results in bacteria present in both the feces and intestinal wall for at least 21 days post-inoculation, and no differences in fecal appearance or cloaca temperature as compared to control birds [26]. The gut is a very low-O_2_ environment [27], whereas *in ovo* embryos, which are commonly damaged by *M. iowae*, are exposed to atmospheric O_2_ due to eggshell permeability [28]. It is intriguing that *M. iowae* infection at aerobic sites, but apparently not the gut, causes disease, leading us to suspect that O_2_ might play a role in regulating expression of *M. iowae* virulence factors, resulting in different outcomes at different body sites.

Differential gene expression regulation exists in a variety of mycoplasma species despite their reduced genomes. The *M. pneumoniae* promoters for acetate kinase and lactate dehydrogenase are strongly induced in the presence of glucose and glycerol, respectively [29]. *M. pneumoniae* lipoprotein genes are also differentially expressed in response to exposure to host cells, H_2_O_2_, and low pH [30]. Multiple forms of regulation occur in *Mycoplasma hyopneumoniae* in response to heat shock [31], iron deprivation [32], H_2_O_2_ treatment [33], and infection of pigs [34]. The means by which most of these regulatory events occur is unknown.

In this study we examined the ability of *M. iowae*, by virtue of its catalase activity, to offer protection to a model host from H_2_O_2_ stress, and to test whether *M. iowae* experiences differential regulation of catalase as well as possible virulence-associated genes in response to changes in O_2_ level. We used *Caenorhabditis elegans* bioassays, developed previously as a model for use with *M. iowae* [13], and reverse transcription-quantitative polymerase chain reaction (RT-qPCR) of selected candidate virulence-associated genes. Our results indicate that catalase contributes to *M. iowae* being protective toward the host against H_2_O_2_-producing organisms, even though catalase activity is reduced under conditions of low O_2_. They also indicate that *M. iowae* grown in low O_2_ results in increased survival of *C. elegans* upon co-incubation as compared with *M. iowae* grown in atmospheric conditions, accompanied by a 5-fold decrease in expression of at least one of the CARDS toxin-like protein genes.

## Materials and methods

### Bacterial strains and growth conditions

Mycoplasma strains used include *M. iowae* serovar K strain DK-CPA, *M. gallisepticum* R_low_, and *M. gallisepticum* R_low_ transformant 56A. For *M. gallisepticum* and for aerobic growth of *M. iowae*, all strains were grown at 37 °C in 175-cm^2^ tissue culture flasks containing 50 mL of SP-4 broth [35] to mid-log phase. Transformant 56A was grown in the presence of 4 μg mL^−1^ tetracycline. SP-4 broth for samples grown in low O_2_ was allowed to equilibrate with a 1% O_2_/99% N_2_ gas mix overnight. Following equilibration, 50 mL of broth was aliquotted into glass bottles that were capped with rubber stoppers in which cultures were grown to mid-log phase at 37 °C.

*Escherichia coli* DH5α was grown in Luria broth (LB) with 100 μg mL^−1^ ampicillin at 37 °C in a shaking incubator. A *Streptococcus pneumoniae* obtained from the Miami University Department of Microbiology stock collection was grown at 37 °C without shaking in brain heart infusion (BHI) broth. *Pseudomonas aeruginosa* strain PAO1, also from the Miami University Department of Microbiology stock collection, was grown at 37 °C with shaking in LB broth.

### Preparation of cell lysates and protein analysis

Fifty- mL cultures of mycoplasma cells were collected by centrifugation at 20 000 × *g* for 20 min and washed three times with cold phosphate-buffered saline (PBS). Cells were resuspended in 1 mL cold PBS containing 1% sodium dodecyl sulfate using a 25-gauge syringe and incubated at 37 °C for 30 min. Cell lysates were stored at −80 °C. Protein concentration in cell lysates was determined using bicinchoninic acid assays (Pierce Biotechnology Inc.).

### Catalase enzyme activity

Catalase activity was measured in whole cell lysates of *M. iowae* using the Amplex Red Catalase Assay kit (Invitrogen). Catalase activity was normalized to total protein concentration in cell lysate samples. Statistical significance of results was calculated using unpaired Student’s *T*-test. Results represent two biological replicates from each condition with four technical replicates each.

### H_2_O_2_ assays

Methods were adapted from Hames et al*.* [36]. For *S. pneumoniae* assays, colonies grown on BHI agar plates were picked and grown overnight in 5 mL of BHI broth at 37 °C without shaking. Cultures were diluted 1:100 in pre-warmed BHI broth and grown to mid-log phase at 37 °C without shaking (OD_620_ = 0.2-0.3). Cells were collected by centrifugation at 10 000 × *g* for 6 min and washed three times in cold HNM buffer (67.6 mM HEPES, pH 7.3, 140 mM NaCl, and 7 mM MgCl_2_. H_2_O_2_ levels were measured using colorimetric test strips (EM Quant, range 0.5-25 mg L^−1^). Four biological replicates were examined. Statistical significance was calculated using unpaired Student’s *T*-test.

To determine H_2_O_2_ production by mycoplasmas alone, 50-mL cultures of mycoplasma cells were grown to mid-log phase. Cells were collected by centrifugation at 20 000 × *g* for 20 min and washed three times in cold HNM buffer. Following resuspension in the same buffer to an OD_550_ = 1.0, aliquots of 1 mL were added to 24-well plates with 500 μM or 1 mM sucrose and incubated at room temperature for 24 h. H_2_O_2_ levels were measured and statistical analysis was performed as described above. Two biological replicates, each with 2 technical replicates, were examined.

To determine H_2_O_2_ production by *S. pneumoniae* when using mycoplasma cells as a source of carbohydrates, both bacteria were grown independently and collected as described above. One-mL aliquots were placed in 24-well plates that contained *S. pneumoniae* at an OD_620_ = 0.05 and mycoplasmas at an OD_550_ = 1.0 with no sucrose. Samples were incubated at room temperature for 24 h. H_2_O_2_ levels were measured and statistical analysis was performed as described above. Three biological replicates were examined.

### *C. elegans* growth conditions

All assays were performed with *C. elegans* strain N2 (Bristol). Nematodes were cultured using standard practices [37]. Briefly, worms were cultured on nematode growth media plates seeded with *E. coli* OP50 as a food source at room temperature on the benchtop.

### *C. elegans* survival assays

Plates containing large, gravid nematodes were treated with hypochlorite solution to obtain sterile eggs using standard procedures [37]. Eggs were hatched overnight in 10 mL of M9 buffer with gentle shaking to obtain L1 larvae. L1 larvae were washed with M9 buffer and aliquotted into 24-well plate wells. The number of live larvae per well (indicated by movement) was counted prior to the addition of samples to a final volume of 1 mL. Plates were incubated at room temperature for the designated time, at which time live nematodes were counted again to measure survival. They were considered dead if no movement was observed in response to shaking or tapping the plate. Following the 24-h incubation period, H_2_O_2_ levels were also recorded with the use of colorimetric test strips as described above. For long-term assays, plates were wrapped in parafilm to prevent evaporation of liquid during the extended incubation time required for the experiment. Long-term assay results represent two biological replicates from each condition with six technical replicates each. Statistical significance of results was calculated using unpaired Student’s *T*-test, with *p* < 0.05 being regarded as significant.

For mycoplasma samples, cells were collected and washed as described for H_2_O_2_ assays. *M. iowae* cells tested alone were resuspended to various OD_550_ values and incubated with *C. elegans* larvae in the presence of the indicated concentrations of H_2_O_2_. Assays examining protection from abiotic H_2_O_2_ were performed with mycoplasma cells resuspended to an OD_550_ = 1.0 in the presence of 8 mg L^−1^ H_2_O_2_. Three biological replicates, each with 3–4 technical replicates, were examined.

For assays with *S. pneumoniae*, bacteria were grown and harvested as described for H_2_O_2_ assays. Samples were added to *C. elegans* larvae with *S. pneumoniae* resuspended to OD_620_ = 0.05 (approximately 1.1 × 10^7^ CFUs), mycoplasmas resuspended to OD_550_ = 1.0 (approximately 1.2 × 10^9^ CFUs for *M. iowae*), and 500 μM sucrose. Three biological replicates, each with 2 technical replicates, were examined.

For assays with *P. aeruginosa*, colonies grown on LB agar plates were picked and grown overnight in 5 mL of LB broth at 37 °C in a shaking incubator. Cultures were diluted 1:100 in pre-warmed LB broth and grown to mid-log phase at 37 °C and 200 rpm (OD_600_ = 0.4). Cells were pelleted by centrifugation at 10 000 × *g* for 10 min and washed three time with cold buffer containing 20 mM L-glutamate, 5 mM K_2_HPO_4_, 5 mM NaH_2_PO_4_, 2 mM MgSO_4_ · 7H_2_O, 0.02 mM FeCl_3_, 12.5 mM glycine, and 50 mM Tris, pH 7.5 [38]. *P. aeruginosa* samples tested alone with worms were resuspended to OD_650_ = 0.1, 0.05, and 0.01 in the same buffer. For assays performed in combination with mycoplasmas, *P. aeruginosa* was used at OD_650_ = 0.1 (approximately 1.1 × 10^8^ CFUs). Mycoplasmas were washed and resuspended in the same buffer as *P. aeruginosa* and used at an OD_550_ = 1.0. Three biological replicates, each with 2–4 technical replicates, were examined.

### Sequence analysis

Predicted amino acid sequences for *M. iowae* CARDS1 and CARDS2 [13] and *M. pneumoniae* CARDS toxin [39] were aligned to one another with BLAST.

### RNA isolation and quantification

RNA was isolated from *M. iowae* cells using TRI reagent (Sigma). Briefly, cells were collected by centrifugation at 20 000 × *g* for 20 min. Following resuspension of cell pellets in TRI reagent, RNA was extracted with chloroform, pelleted with isopropanol, and washed with 75% ethanol. RNA was resuspended in 100–300 μL DEPC-treated water and stored at −20 °C. To eliminate DNA contamination, samples were treated with DNase I (Invitrogen or QIAGEN) according to the manufacturer’s instructions. RNA was then cleaned up using the RNeasy Mini Kit (QIAGEN). Elimination of DNA contamination was confirmed by PCR with *glpF* primers (Table 1). RNA quality was determined by analysis on an RNA Pico Chip (Agilent Technologies) using a Bioanalyzer 2100 (Agilent Technologies).

**Table 1:** **Primers used for qPCR experiments**

**Primer Name**	**Target**	**Sequence (5′-3′)**	**Tm (°C)**
MI16Sleft(qPCR)	16S rRNA gene	CGCAAGACTCACGAGCTTAT	54.6
MI16Sright(qPCR)		GGTACAAACTGTCGCAAACC	54.4
MIcards1left(qPCR)	*cards1 (P271_571)*	TGGGTAGAAGCACAGACGTT	56.1
MIcards1right(qPCR)		ACTCATCTGCATCTGGGTCA	55.8
MIglpFleft(qPCR)	*glpF (P271_673)*	ATCTAGCATGATGGGTGGCG	57.3
MIglpFright(qPCR)		TGTCCAAACATTGCTCCTGT	54.7
MIkatEleft(qPCR)	*katE (P271_534)*	CGTGTAGTTCATCGAAAAGGTG	54.6
MIkatEright(qPCR)		CTTCCAGCTTCACCACCAAC	56.3
MIsodleft(qPCR)	*sodA (P271_491)*	ACACAAAGCATCACCAAGCT	55.2
MIsodright(qPCR)		TGATTGTGATGACCTCCACCA	56.1

### Reverse transcription (RT)

RT was carried out with the Verso cDNA Synthesis Kit (Thermo Scientific) according to the manufacturer’s instructions. One hundred ng of RNA was used as starting material and random hexamers were used as primers in reactions with a final volume of 20 μL. Reactions were incubated at 70 °C for 5 min, 42 °C for 30 min, 95 °C for 2 min, and then chilled on ice. cDNA synthesis was confirmed by PCR with *glpF* primers (Table 1). Controls were performed to exclude the possibility of DNA contamination in which the same reactions were performed with water substituted for Verso Enzyme Mix and RT Enhancer. Control reactions were treated and stored identically to other samples.

### Genomic DNA isolation and standard generation

Genomic DNA was isolated from a 50-mL culture using the QIAamp® DNA Mini Kit (QIAGEN). To improve quantification, plasmids were constructed that contained a copy of each gene target. Plasmids were constructed from PCR products amplified from *M. iowae* genomic DNA using gene-specific primers (Table 2) that were ligated into pCR®2.1 (Invitrogen) and transformed into competent *E. coli* DH5α. Plasmids were isolated from clones with the Zyppy™ Plasmid Miniprep Kit (Zymo Research). Insertion was confirmed with digestion by *Eco*RI and sequence was confirmed using vector-specific primers M13F and M13R (Table 2). Gene copy numbers were calculated using the concentration of each plasmid assuming 1.096 × 10^−12^ g bp^−1^ [40]. Standard curves were generated from ten-fold dilutions of DNA with known copy numbers and were analyzed by qPCR in triplicate.

**Table 2:** **Primers used for construction and sequencing of qPCR standards**

**Primer name**	**Target**	**Sequence (5′-3′)**
MIcards1up	*cards1*	ATCGTCTGGTGCATATGCAACAGC
MIcards1down		ATCGGCTCATGCAAGTGTTGCAGC
MIglpFup	*glpF*	ATCGGTAGTGCTTTTGCACTACAC
MIglpFdown		ATCGCTTCCAATGATTCCACCTCC
MIkatEupSalI	*katE*	ATCGGTCGACAAATGCTGCAACAGCTGCAC
MIkatEdownSalI		ATCGGTCGACTAAACACAAAATTTGATTTAATCAAAATTCATG
MIsodup	*sodA*	ATCGGAACTGGGCCAACAAATGAC
MIsoddown		ATCGACTTCACATGTCAGTTAGGG
M13Forward	Sequencing	GTTGTAAAACGACGGCCACT
M13Reverse		CAGGAAACAGCTATGACC

### RT-quantitative PCR (qPCR)

Each RT-qPCR reaction was done in at least triplicate using two biological replicates. Reactions were performed with PerfeCTa® SYBR® Green Supermix (Quanta Biosciences), 1 μL of cDNA or DNA template (5 ng), and 300 nM gene-specific primers (Table 1) in a final volume of 25 μL. Amplification conditions were 5 min at 95 °C for 3 min followed by 40 cycles of 10 s at 95 °C and 45 s at 52 °C. To determine the melting temperature and PCR product specificity, a melting curve was obtained after every run by heating from 50 °C (2 °C below T_a_) to 95 °C. Primer specificity was determined by melting curve analysis and agarose gel electrophoresis of PCR products. Controls with no template or reverse transcriptase were included for each sample during each run. Runs were performed using the CFX Connect (Bio-Rad) and analysis was performed using Bio-Rad CFX Manager 3.0 software (Bio-Rad). The 16S rRNA gene was used for normalization. Statistical analysis of results was calculated using unpaired Student’s *T*-test. MIQE guidelines were followed for performing and reporting experiments [41].

## Results

### Catalase confers host protection from peroxigenic bacteria

*M. iowae* has an active catalase protein, enabling reduction of H_2_O_2_ by this organism [13]. *C. elegans* is a well-established model for the study of H_2_O_2_-mediated bacterial pathogenicity [1,2,42], and has recently been adapted for use with mycoplasma cells [13]. *M. gallisepticum* naturally lacks catalase activity, but *M. gallisepticum katE* transformant 56A, which produces *M. iowae* catalase, produces less H_2_O_2_ and kills fewer *C. elegans* larvae in the presence of the peroxigenic molecule glycerol than wild-type *M. gallisepticum* R_low_, suggesting a role for catalase in protection from killing [13]. Incubation with *M. iowae* at OD_550_ = 0.01 increased survival of *C. elegans* from 30% in the absence of bacteria to 50% (not shown), a reasonable set of conditions for subsequent assays.

**Figure 1:**
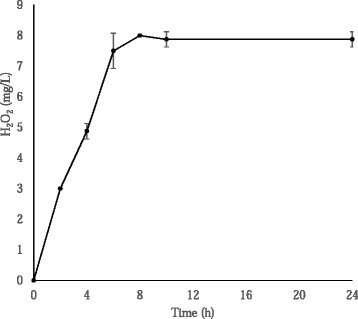
**H**_**2**_ **O**_**2**_ **accumulation by** ***S. pneumoniae*** **.** Accumulation of H_2_O_2_ over time by *S. pneumoniae* at an OD_620_ of 0.05 in the presence of 500 μM sucrose. Error bars, SD.

**Figure 2:**
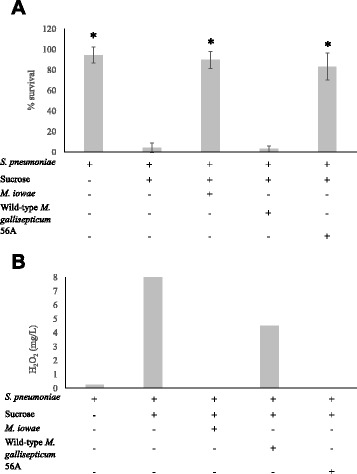
**Host protection from** ***S. pneumoniae*** **-produced H**_**2**_ **O**_**2**_ **by catalase-positive mycoplasmas.** Survival of *C. elegans* **(A)** and amount of H_2_O_2_ remaining **(B)** at 24 h upon incubation with *S. pneumoniae* at OD_620_ = 0.05 in the presence of 500 μM sucrose. Experiments were performed in triplicate. Results shown are from one representative experiment (B). Error bars, SD. *, statistically significantly different from *S. pneumoniae* alone with 500 μM sucrose (*p* < 0.05).

In the vertebrate host, bacteria can come into contact with multiple sources of H_2_O_2_, including host immune defense mechanisms and other microbial pathogens [1,2,43]. To mimic more closely conditions of H_2_O_2_ exposure in the host and test whether *M. iowae* was protective under these conditions, *C. elegans* survival assays were performed with the use of H_2_O_2_ produced continuously by a biotic source. *S. pneumoniae* is a bacterial pathogen that produces H_2_O_2_ from a variety of substrates including sucrose [44-46], a carbohydrate that *M. iowae* and *M. gallisepticum* are unable to metabolize [17]. When *S. pneumoniae* resuspended to OD_620_ = 0.05 was incubated with 500 μM sucrose at room temperature for 24 h, H_2_O_2_ accumulated rapidly, reaching maximum levels after 8 h of incubation, at which point H_2_O_2_ levels remained constant for the duration of the 24 h period (Figure 1). *M. iowae* or wild-type *M. gallisepticum* resuspended to OD_550_ = 1.0 and incubated under the same conditions produced less than 1 mg L^−1^ H_2_O_2_, respectively, and the same outcome occurred when mycoplasma cells were co-incubated with *S. pneumoniae* in the absence of sucrose (data not shown; see Figure 2B). Based upon these results, *C. elegans* survival assays were performed with *S. pneumoniae* at OD_620_ = 0.05, mycoplasmas at OD_550_ = 1.0, and 500 μM sucrose.

When incubated with *S. pneumoniae* alone, approximately 6% of *C. elegans* larvae survived for 24 h in the presence of sucrose (Figure 2A), correlating with an accumulation of 8 mg L^−1^ H_2_O_2_ (Figure 2B). In the absence of sucrose, very little H_2_O_2_ accumulated (Figure 2B) and almost all *C. elegans* larvae survived upon incubation with *S. pneumoniae* (Figure 2A), demonstrating that under these conditions the toxicity was associated primarily with H_2_O_2_. Inclusion of wild-type *M. gallisepticum* with *S. pneumoniae* and sucrose did not significantly alter the amount of *C. elegans* survival as compared to *S. pneumoniae* alone with sucrose (Figure 2A). However, H_2_O_2_ accumulation decreased to 4.5 mg L^−1^ (Figure 2B), possibly because of loss of H_2_O_2_ upon reaction with the high number of *M. gallisepticum* cells in suspension. On the other hand, inclusion of catalase-producing *M. iowae* or *M. gallisepticum* transformant 56A resulted in significantly increased amounts of *C. elegans* survival (Figure 2A) and no detectable H_2_O_2_ (Figure 2B). Taken together, these results suggest that catalase enables *M. iowae* to offer protection from H_2_O_2_ being continually produced by other organisms.

**Figure 3:**
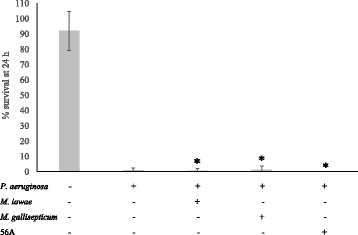
**Absence of host protection from** ***P. aeruginosa*** **by catalase-positive mycoplasmas.** Survival of *C. elegans* at 24 h upon co-incubation with *P. aeruginosa* at OD_650_ = 0.1. Experiments were performed in triplicate. Error bars, SD. *, no statistically significant difference from *P. aeruginosa* alone (*p* > 0.05).

Reasoning that protection by *M. iowae* is due to catalase and therefore might be specific to H_2_O_2_-mediated stress, *C. elegans* survival assays were repeated with a non-H_2_O_2_ producing pathogen. *P. aeruginosa* PAO1 produces the toxic molecule HCN [47], to which *C. elegans* larvae are highly susceptible; incubation with this bacterial strain typically results in complete killing of larvae [48,49]. At OD_650_ = 0.1, *P. aeruginosa* caused almost complete killing of larvae at 24 h (data not shown). Under these conditions, co-incubation of *C. elegans* with *P. aeruginosa* and any of the mycoplasma strains resulted in no difference in the survival of larvae at 24 h (Figure 3).

### O_2_-dependent changes in catalase activity and long-term survival of *C. elegans*

**Figure 4:**
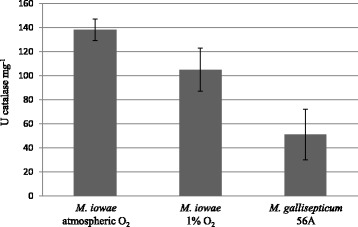
**Catalase activity in** ***M. iowae*** **and** ***M. gallisepticum*** **transformed with** ***katE*** **.** Extracts from cells grown under atmospheric conditions or in 1% O_2_ were assayed for catalase activity as described in Material and methods. Error bars, SD.

*M. iowae* lives in both aerobic and reduced-oxygen environments in its natural host, but its pathogenicity appears to be limited or non-existent in the gut, where it accumulates but where O_2_ is low. Therefore, we explored whether *M. iowae* grown under low O_2_ conditions might be less toxic to host cells. Interestingly, catalase activity of *M. iowae* significantly decreased by 24% (*p* < 0.05) in low O_2_ (Figure 4), suggesting that O_2_ exposure has an impact on catalase activity in *M. iowae. M. gallisepticum* 56A, which offered protection comparable to that of *M. iowae* [13] had 63% less catalase activity (*p* < 0.05) than *M. iowae* under atmospheric conditions (Figure 4). Therefore the decreased catalase activity in *M. iowae* cells grown in 1% O_2_ is unlikely to result in a significant decrease in protection.

**Figure 5:**
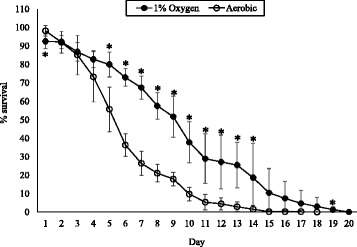
**Long-term survival of** ***C. elegans*** **when fed** ***M. iowae*** **grown aerobically or in the presence of 1% O**_**2**_ **.** Error bars, SD. *, statistically significantly different from aerobically-grown *M. iowae* (*p* < 0.05).

Whereas diffusible molecules such as H_2_O_2_ are responsible for damage to *C. elegans* within the first 48 h of incubation, the impact of toxins becomes apparent after 48 h as bacteria are able to establish an infection within the nematode gut [2]. To examine differences in the ability of *M. iowae* to cause damage to its host, long-term *C. elegans* assays were performed with bacterial cells grown under different O_2_ concentrations. It is important to consider that the *C. elegans* assay conditions are carried out in a buffer, preventing mycoplasma growth, and at room temperature, which is well below the temperature range at which *M. iowae* can grow. It is therefore very likely that by the end of the incubation with *C. elegans*, *M. iowae* cells have no more catalase or putative toxins than they had at the beginning. A repeated measures ANOVA revealed significant differences between the two groups [*F*(1,17) = 27.39, *p* < 0.05]. Cells grown aerobically caused nematodes to die much more quickly, with 50% survival achieved after approximately 5 days as compared to approximately 9 days with cells grown in the presence of 1% O_2_, and death of all larvae by 15 days with aerobically-grown *M. iowae* cells as opposed to 20 days with low O_2_-grown mycoplasmas (Figure 5). These data suggest that *M. iowae* cells are less harmful in low-O_2_ environments.

### O_2_ regulation of potential *M. iowae* virulence genes

**Figure 6:**
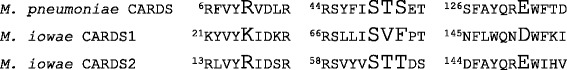
**Alignment of CARDS toxin-like sequences from** ***M. pneumoniae*** **and** ***M. iowae*** **.** Residues generally conserved in other ADP-ribosylating toxins are bolded and enlarged.

Because it is unlikely that a relatively small O_2_-dependent decrease in *M. iowae* catalase activity was related to increased survival of *C. elegans*, we hypothesized that *M. iowae* genes more directly linked to virulence experienced O_2_-dependent regulation. Candidate virulence-associated genes were selected for analysis of regulation of expression by O_2_. Transcription of the potential virulence factors *glpF* and *cards1* was examined as well as *katE* and another antioxidant enzyme, *sodA. glpF*, which allows uptake of glycerol for production of H_2_O_2_, was chosen as a representative of the presumptive glycerol catabolism operon that includes *glpK* and *glpO*. Additionally, *M. iowae* serovar K has two copies of genes homologous to *M. pneumoniae* CARDS toxin [13]. We examined the predicted amino acid sequences for the proteins encoded by these genes. There are three common motifs shared by many ADP-ribosylating toxins: a conserved arginine for NAD^+^ binding, a serine-threonine-serine motif to maintain structural integrity of the NAD^+^ binding site, and a catalytic glutamate [20]. These amino acids are all present in *M. pneumoniae* CARDS toxin, which has ADP-ribosyltransferase activity [20]. When both *M. iowae* CARDS toxin homologs were examined (named CARDS1 and CARDS2), some deviations were observed (Figure 6). CARDS2 has a conservative change of the second serine residue in the STS motif to a threonine. CARDS1 contains multiple changes, with substitutions of like charge at the conserved arginine and glutamate residues and less conservative substitutions for two of the three amino acids in the serine-threonine-serine motif. These *M. iowae* homologues have 28% and 25% identity, respectively, to the *M. pneumoniae* CARDS toxin sequence. Each *M. iowae* gene also has 99% identity to its respective homolog in *M. iowae* serovar I strain 695 [50]. *cards1* was chosen despite the greater degree of disparity from *M. pneumoniae* CARDS toxin since only 20 bases separate *cards1* and *cards2*, likely making both genes transcriptionally linked.

To examine transcript levels in *M. iowae* cells grown under different O_2_ concentrations, qPCR was performed (Table 3). Expression of 16S rRNA was used as a control. The putative toxin gene *cards1* and the catalase gene *katE* underwent statistically significant down-regulation in *M. iowae* cells grown in the presence of 1% O_2_, with decreases of 4.9- and 5.4-fold, respectively (Table 3). In contrast, *glpF* and *sodA* did not exhibit significant changes in expression in response to O_2_ availability. Taken together, these data suggest that *M. iowae* undergoes differential regulation of select genes in response to low O_2_ conditions. The reduction in *cards1* expression is consistent with reduced pathogenicity in the gut.

**Table 3:** **Differences in gene expression as determined by qPCR in response to growth in the presence of 1% O**_**2**_

**Gene**	**Fold-change of down-regulation in response to 1% O**_2_^*^
*cards1*	4.93 ± 0.78^†^
*glpF*	1.67 ± 0.88
*katE*	5.37 ± 2.60^†^
*sodA*	1.35 ± 1.06

^*^Average ± SD.

^†^ *p* < 0.05 compared to aerobic expression as determined by unpaired Student’s *T*-test.

## Discussion

Both genome sequences currently available for *M. iowae* [13,50] reveal the presence of a gene for catalase, an H_2_O_2_-degrading enzyme absent from all other published mycoplasma genomes, which produces an active protein [13]. Bacteria can encounter exposure to H_2_O_2_ from a variety of different sources, including other bacterial pathogens as well as the host immune response [1,2,43]. Bacteria bearing enzymes that detoxify ROS, including catalase, have been demonstrated to provide benefit to animal hosts in the context of probiotics [9]. Our results support a model wherein protection by *M. iowae* is specific to catalase-mediated reduction of H_2_O_2_ stress, but other mechanisms are also possible. Catalase is specifically implicated in this protection because *M. gallisepticum*, which does not normally have catalase activity, becomes protective upon transformation with *M. iowae katE* [13], despite 2.7-fold less catalase activity in the *M. gallisepticum* transformant 56A as compared with *M. iowae*.

Host-associated bacteria live in the presence of a consortium of other microorganisms, some of which may be H_2_O_2_ producers. We used *S. pneumoniae* as a model pathogen that produces H_2_O_2_ as an important virulence factor that not only can cause damage to host cells but has also been studied in the context of *C. elegans* [51-53]. Furthermore, *S. pneumoniae* can inhibit the growth of other bacterial competitors by virtue of its H_2_O_2_ production [54]. The ability of *M. iowae* to protect against H_2_O_2_-mediated damage caused by *S. pneumoniae*, albeit not against other kinds of damage such as that caused by *P. aeruginosa*, suggests that *M. iowae* could be beneficial to its host provided it is not also producing molecules harmful to its host.

*M. iowae* causes damage at multiple sites throughout the body of its natural poultry hosts, including legs, joints, air sac, and feathers [14-16]. However, despite many accounts of detection in and isolation from the gut, no clear-cut reports of disease at this site due to *M. iowae* have been documented [23-26]. Because low O_2_ concentration is a hallmark of the gut environment that distinguishes it from other sites at which *M. iowae* causes damage and disease [27], we examined the impact of growth in 1% O_2_ on *M. iowae* with regard to activities that might be associated with both disease and protection. Our finding that *M. iowae* has an ability to prolong survival when fed to *C. elegans* larvae following growth in 1% O_2_ supports the notion that *M. iowae* is not harmful in the gut and might in fact be a beneficial component of the gut microflora. Significantly, at the transcriptional level, this environment causes a significant down-regulation in not only the gene encoding catalase, but also a gene encoding a homolog of CARDS toxin, a causative agent of host damage by *M. pneumoniae* [20,55] and *M. penetrans* [56]. These data suggest that *M. iowae* undergoes differential regulation of select genes in response to growth in low O_2_ environments.

The down-regulation of *katE* in 1% O_2_ could be a response to a decreased threat of damage from ROS. Despite this reduction, it is important to consider that the catalase activity in low O_2_-grown *M. iowae* cells is still 2.1-fold greater than the amount present in *M. gallisepticum* transformant 56A, which itself still confers significant protection of *C. elegans* exposed to H_2_O_2_ [13]. Therefore, the reduction in *M. iowae* catalase activity in a gut-like environment is not necessarily associated with decreased protection. It is possible that production of catalase facilitates colonization of the gut by *M. iowae*, in parallel with *Campylobacter jejuni*, which expresses an active catalase that is important for colonization of the poultry intestinal tract [57]. The degree of down-regulation of *katE* at the transcription level does not closely match the change in catalase activity in whole cell lysates, with a smaller decrease observed in the latter. The high level of catalase activity might not decrease linearly as the concentration of catalase decreases.

The down-regulation of *cards1*, a homolog of CARDS toxin, which is likely accompanied by similar down-regulation of the very closely linked *cards2*, may be associated with decreased pathogenicity of *M. iowae* in the gastrointestinal tract. The amount of CARDS toxin produced correlates positively with the amount of host damage caused by *M. pneumoniae* [55]. Differential expression of toxins in different environments is well-established in diverse bacteria, including clostridia [58], *Vibrio cholerae* [59], and cyanobacteria [60]. It is conceivable that the environment of the intestine is sufficiently nutritious for *M. iowae* that severe damage to host cells is unwarranted, favoring a strategy in which virulence factors may be used principally in environments in which nutrients are less readily available. In this model, low O_2_ provides a cue to *M. iowae* that it is in the gut, where it can conserve energy by down-regulating virulence genes, including those encoding the CARDS toxin-like proteins. The similar down-regulation of both *cards1* and *katE* transcript levels when grown in the presence of 1% O_2_, on the order of 5-fold, raises the possibility that a common regulatory mechanism may be acting on both genes, but further work is necessary to elucidate such a mechanism.

## Abbreviations

ROS: Reactive oxygen species
IBD: Inflammatory bowel disease
RT-qPCR: Reverse transcription-quantitative polymerase chain reaction
BHI: Brain heart infusion
PBS: Phosphate-buffered saline.
